# Factors Related to Self-Confidence to Live Alone in Community-Dwelling Older Adults: A Cross-Sectional Study

**DOI:** 10.1186/s12877-021-02214-w

**Published:** 2021-05-04

**Authors:** So Im Ryu, BeLong Cho, Sun Ju Chang, Hana Ko, Yu Mi Yi, Eun-Young Noh, Hye Ryung Cho, Yeon-Hwan Park

**Affiliations:** 1 College of Nursing, The Research Institute of Nursing Science, Seoul National University, Seoul, 103, Daehak-ro, Jongno-gu, Seoul, 03080 Republic of Korea; 2 Department of Family Medicine, College of Medicine, Seoul National University, Seoul, 103, Daehak-ro, Jongno-gu, Seoul, 03080 Republic of Korea; 3 Institute on Aging, Seoul National University College of Medicine, 71 Ihwajang-Gil, Jongno-gu, Seoul, 110-810 Republic of Korea; 4 College of Nursing, Seoul National University, Seoul, 103, Daehak-ro, Jongno-gu, Seoul, 03080 Republic of Korea; 5 College of Nursing, Gachon University, 191 Hambakmoero, Yeonsu-gu, Incheon, 21936 Republic of Korea; 6 College of Nursing, Kyungnam College of Information and Technology, 45 Jurye-ro, Sasang-gu, Busan, 47011 Republic of Korea

**Keywords:** Ageing, Independent living, Self-concept, Social isolation, Depression, Safety

## Abstract

**Background:**

Many older adults prefer to live alone in their own homes, with age-related issues in physical movement, regardless of their cultural background. Importantly, however, to identify the features of successfully ageing in place (AIP), and foster independent living among these individuals, this study explored their level of self-confidence to live alone and its related factors.

**Methods:**

We conducted a cross-sectional study using secondary data from an earlier study with older adults living alone in South Korea recruited by convenience sampling methods (*N* = 936, mean age = 77.1 years, 76.1% female). Data regarding the general, health-related, and social characteristics as well as self-confidence to live alone were collected via face-to-face interviews in 2019. Self-confidence to live alone was measured with a numeric rating scale of 0 to 10.

**Results:**

The average self-confidence score to live alone was 6.59. A regression analysis showed that mould exposure at home, depression, emergency department visits, and loneliness hinder self-confidence to live alone. Meanwhile, such self-confidence was facilitated by independency in instrumental activities of daily living (IADL), interactions with family members, social service utilisation, and social support.

**Conclusions:**

This study suggests that healthcare providers need to consider the importance of self-confidence to live alone and influencing functional, mental, social, and environmental factors to promote quality of life as well as successful AIP for older adults living alone. Further, self-confidence to live alone could be a new practical index in the field of health and ageing to screen the successful AIP of older adults living alone.

## Background

The number of older adults living alone has risen and is expected to increase, particularly in Western and developed countries, including South Korea [1, 2]. Notably, these individuals are a vulnerable group that can face serious challenges to successfully ageing in place (AIP) because they tend to be socially isolated, feel lonelier [3], have poor health status [4], and lower life satisfaction [5]. Nevertheless, a majority of them strongly prefer to live in their own home, even in cases of age-related issues in physical movement, regardless of their cultural background [6, 7].

AIP has emerged as a key concept in the field of gerontology, reflecting an increase in the ageing population, and their living preferences [8]. It is defined as ‘meeting the desire and ability of people, through the provision of appropriate services and assistance, to remain living relatively independently in the community in his or her current home or an appropriate level of housing’ [9]. As many researchers have asserted a positive impact on self-identity, autonomy, and well-being, AIP has become an important strategy to overcome the challenges that we encounter [10, 11]. However, recent studies have pointed out that all stakeholders need to be alert about the ‘stuck-in-place’ issues that accompany living with a shortage of financial, physical, and social resources necessary to live independently [12].

For older adults living in the community, independent living means to live in their own homes with a sense of control and freedom of decision and movement [13]. Sense of control has been considered a ‘status’ goal in many studies [14]. However, as successful AIP is an eventual process of balancing and adapting to both personal and environmental changes in the way of ageing [15], previous concepts such as sense of control are limited to reflecting dynamic interactions between a person and their environment. A previous qualitative study focussing on the concept of AIP emphasised that it is a process rather than a constant state, and studied the perception of one’s ability as a feature of a successful AIP [16]. In addition, previous qualitative studies have demonstrated that a salient attribute of a person ageing successfully is their faith and trust to live independently and adapting to any circumstances even with physical discomfort; this can be defined as self-confidence [17–19]. Therefore, successful AIP can be considered as ageing with confidence in one’s ability to live independently with a sense of control [17]. However, self-confidence to live alone, as a pioneering concept for AIP, has not yet been explored. Thus, we need to comprehensively examine the factors that influence this, along with the accumulated knowledge of factors for AIP and independent living.

According to previous studies, regarding demographic characteristics, women and old people (≥75 years old) are less likely to AIP [17], while having religion, a higher income, independent earning, higher educational level, and satisfaction with the condition of housing, facilitates AIP [6, 20]. Many previous studies related to residential spaces have been concerned with ‘age-friendly modification’ such as basic housing facilities [21]. In fact, the World Health Organization [22] reported the prevalence of indoor dampness estimated at 10–50% worldwide. In addition, 20–30% of the older adults who were at risk of poverty in Europe, reported experiencing leaky roofs, dampness, and broken windows or rotten frames, as was the case in South Korea [23, 24]. Thus, it is necessary to explore the effect of mould exposure and wall cracks at home on individuals’ self-confidence to live alone.

Daily living difficulties like health-related characteristics, disabilities, and other instrumental activities hinder independence negatively and affect independent living and AIP [25, 26]. In addition, those with more chronic conditions and depressive symptoms are less likely to live independently [6, 26]. According to a qualitative study that explored the experience of AIP by interviewing older women with physical discomfort living alone, ‘falling’ was one of the biggest challenges they faced in daily living [19]. Due to a decline in physical, sensory, and cognitive functions, older adults experience the highest risk of fall-related deaths or severe injuries [27], and approximately 33.7% injured by falls, visit the emergency department (ED) [28]. In particular, older adults who live alone experience helplessness because there is no one to help them in the event of a fall, which leads to feelings of fear and lack of safety [29]. Despite the emphasis on health-related safety for AIP, it has rarely been addressed to the best of our knowledge [30].

Concerning social characteristics, participation in social activities [19, 21]; interaction with friends, neighbours, and family [21]; and social support, positively affects AIP [17]. However, loneliness affects AIP negatively in older adults [17]. Even though the importance of community-based services has been emphasised [10], a longitudinal study demonstrated that older adults who used community-based services were less likely to age in place [6]. Thus, the effect of public service on older adults living alone needs to be further explored.

Inferring from previous studies, some general characteristics (religion, income, educational level, condition of house, participation in economic activity), social networks, and social support can promote self-confidence to live alone, whereas other general characteristics (female, old age), decreased function (disability, IADL), physical condition (number of comorbidities), mental health (depressive symptoms), and loneliness can hinder it. Further exploration is needed with regard to wall cracks and mould exposure at home, falls, ED visits, and public service utilisation.

Therefore, it is necessary to identify the factors influencing the self-confidence to live alone, comprehensively, with respect to general, health-related, and social characteristics.

### Purpose

This study aimed to explore the extent of self-confidence to live alone and related factors in older adults living alone.

## Methods

### Design

This is a cross-sectional study using secondary data from a project funded by the Ministry of Health & Welfare, Republic of Korea (Grant number: HI18C1284). The project aimed to identify service needs and develop a community-based integrated service for older adults living alone.

### Participants

The participants of this study were South Korean older adults living alone in S*city who participated in the original project’s second-year cohort study. Among the participants (*n* = 1041) in the original study, a total of 936 participants (aged 67–96 years) without severe cognitive impairment (MMSE ≤17), who could be evaluated for the ability of instrumental activity of daily living, and had no acute mobility problems due to facture (including arthrosis or spondylopathy) or severe disease surgery within the two months prior to the study, were chosen as the sample for this study.

Power analysis was conducted using the G*power 3.1 programme. To identify factors influencing the self-confidence to live alone, effect size was calculated using linear multiple regression (*α* = .05, *β* = .05, effect size *f*^*2*^ = .06), as described in a previous study (*Adj-R*^*2*^ = .41) [26]. To secure power, at least 546 participants were needed under these conditions. Therefore, this study satisfied the minimum number of participants to secure power.

### Measurements

#### General characteristics

Age, sex, educational level, religion, personal income, participation in economic activity, wall cracks, and mould exposure at home, were evaluated to assess the participants’ general characteristics. While age and personal income were measured as continuous variables, sex, educational level, religion, participation in economic activity, wall cracks, and mould exposure at home were measured as categorical variables. Personal income was categorised based on the average income of older adults in Korea (980,000 KRW/month) in 2017 [7].

#### Health-related characteristics

To assess the health-related characteristics of the participants, functional, physical and mental health, fall history, and ED visits were evaluated.

##### Functional health

Disability was checked as a binomial variable (yes/no) based on whether it was diagnosed and registered in the government grading system. Instrumental activities of daily living were evaluated with the Korean Instrumental Activities of Daily Living (K-IADL) Scale, which has been widely used in Korea [31]. The K-IADL comprises 10 items for evaluating older adults’ functions, such as grooming, housework, and preparing meals. All items are self-reported on a 3-point Likert scale with the following options: independent, partially dependent, and dependent. The total score ranges from 10 to 30, with higher scores indicating greater independence in instrumental activities of daily living. Respondents were categorised into the three groups: independent (all answers independent), partially dependent (at least one answer partially dependent), and dependent (at least one answer dependent) based on their report [32]. The Cronbach’s alpha value was .94 in the validity-identifying study [33]; in the current study, it was .81.

##### Physical health

The number of comorbidities as a continuous variable was counted against a specific list of diseases. The list was compiled based on the ‘2017 survey of living conditions and welfare needs of Korean older persons’ [7] and modified by the authors to include some additional diseases.

##### Mental health

The Korean version [34] of the Geriatric Depression Scale Short Form [35] was used to evaluate depressive symptoms. This is a 15-item scale, and all items are self-reported as binomial variables (yes/no); total scores range from 0 to 15, with higher scores indicating more depressive symptoms. The scores are classified into the following categories: normal (≤ 4), depression (≥ 5). In both the Korean versions of the scale [34] and in the current study, Cronbach’s alpha was .88.

##### Falls

Falls were measured as an ordinal variable using a single question: ‘How many times have you fallen in the last year? (i.e. coming to fall on the ground or floor or other lower level)’ [27].

##### ED visits

ED visits were measured as an ordinal variable with a single question: ‘How many times have you visited the ED in the last year?’

#### Social characteristics

##### Social network

The social network measured the frequency of participation in social activities, and interactions with family members and neighbours (friends) by referring to the ‘Survey on the current status of older adults living alone’, which is used to select targets for ‘Care services for older Koreans’ [36]. When analysed, these were converted to binary variables based on previous studies. Social interactions and interactions with family and neighbours (friends) were categorised against the baseline of three times a week [37] and monthly contact, respectively [38].

##### Public service utilisation

The utilisation of public services was identified based on the items of the ‘Survey on the current status of older adults living alone’ [31] by adding and modifying some items frequently used by older adults living alone in Korea (e.g. basic or comprehensive care services for older Koreans).

##### Social support

Enhancing Recovery in Coronary Health Disease (ENRICHD) Social Support Instrument (ESSI) [39], which was translated into Korean, was used [40]. This 6-item instrument was self-reported as a binomial variable (yes/no). Total scores range from 0 to 6, with higher scores indicating higher social support. Scores can be classified into three categories: good score = 6, fair score = 4 to 5, and poor score ≤ 3. Cronbach’s alpha of the Korean version of the scale was .84; in the current study, it was .78.

##### Loneliness

The Korean version [41] of the revised UCLA Loneliness Scale [42] was used to evaluate the level of loneliness. The scale comprises 20 self-reported items with a 4-point Likert scale (1 = *never* to 4 = *often*). Total scores range from 20 to 80, with higher scores indicating greater loneliness. Scores can be classified into four categories: low (≤ 34), moderate (35 to 49), moderately high (50 to 64), high (≥ 65) [43]. Cronbach’s alpha values for the Korean version of the scale and the current study were .93 and .91, respectively.

#### Self-confidence to live alone

Self-confidence to live alone was measured using a single question: ‘What is your current level of self-confidence to live alone?’ Participants responded with a numeric rating scale (NRS) from 0 for ‘no confidence’ to 10 for ‘very confident’.

### Data collection

The original data were collected through face-to-face interviews by trained research assistants from August 12 to 23, 2019 at health or welfare centres in S* City, South Korea. All research assistants were trained in the objectives, research tools, data collection methods, instructions for questionnaires, and ethical considerations of the study for 2 h or more. The researchers received coded data, which were used for secondary data analysis.

### Data analysis

All statistical analyses were performed using the SPSS version 22 (IBM Corp., Armonk, N.Y., USA) with *p* < .05 as the significance level. Participants’ self-confidence in living alone and all characteristics including general, health, social, and health-related safety were analysed using descriptive statistics (e.g. means, standard deviations, numbers, percentiles, ranges). To determine the differences in self-confidence and the relationship between participant characteristics, independent *t*-tests and Pearson’s correlation coefficients were used for categorical and continuous variables, respectively. To evaluate the predictors of self-confidence to live alone, hierarchical multiple linear regression analyses were performed. We chose predictors with *p* < .05 in the bivariate analyses for the regression model.

## Results

### Participants’ self-confidence to live alone and general characteristics

Among the 936 participants, the average score on self-confidence to live alone was 6.59 on the scale from 0 to 10 (Table 1). The mean age was 77.1 years, 76.1% were female, and more than half of the participants (68.1%) had an educational level below elementary school and followed a religion (66.3%). The participants’ average personal income was approximately 613,000 KRW, mostly lower than 980,000 KRW, which is the average income of older adults in South Korea. Further, 65.9% of the participants were not involved in economic activity, 20.4% had cracks in the walls, and 29.8% had mould in the house.

**Table 1:** Self-confidence to live alone and general characteristics of participants (*N* = 936)

Variables	Categories	***n*** (%)	Mean ± ***SD***	Range
Self-confidence to live alone			6.59 ± 3.01	0–10
**General characteristics**				
Age (years)	65–74	315 (33.7)	77.1 ± 5.44	67–96
	75–84	533 (56.9)		
	≥ 85	88 (9.4)		
Sex	Male	224 (23.9)		
	Female	712 (76.1)		
Educational level	Uneducated	333 (35.6)		
	Elementary school	304 (32.5)		
	Middle school	143 (15.3)		
	High school	121 (12.9)		
	≥ College	35 (3.7)		
Religion	Yes	621 (66.3)		
	No	315 (33.7)		
Personal income	< 980,000 KRW/month	837 (89.4)	612,511 ± 398,021	0–6,000,000
	≥ 980,000 KRW/month	99 (10.6)		
Participation in economic activity	≥ 3–4 times/week	241 (25.7)		
	≥ 1–2 times/week	73 (7.8)		
	≥ 1–2 times/month	5 (0.5)		
	None	617 (65.9)		
Wall cracks at home	Yes	191 (20.4)		
	No	745 (79.6)		
Mould exposure at home	Yes	279 (29.8)		
	No	657 (70.2)		

*SD* Standard deviation, *IADL* Instrumental activities of daily living, *KRW* Korean Won

### Participants’ health-related and social characteristics

Table 2 shows the health-related and social characteristics of the participants. A total of 20% of the participants had disabilities, and 81.4% performed IADL independently. Most participants (95.6%) had one or more diseases with an average of approximately four diseases in total, and more than half of the participants had depressive symptoms. Additionally, 26.1% of the participants reported experiencing one or more falls, of which 5.7% experienced three or more falls. Approximately 11.2% of the participants had visited the ED within the last year, and approximately 1% reported that they had visited the ED more than three times.

**Table 2:** Health-related and social characteristics of participants (*N* = 936)

Variables	Categories	***n*** (%)	Mean ± ***SD***	Range
**Health-related characteristics**				
Disability	Yes	187 (20.0)		
	No	749 (80.0)		
IADL	Independent	762 (81.4)	10.49 ± 1.54	10–24
	Partially dependent	148 (15.8)		
	Dependent	26 (2.8)		
Number of comorbidities	None	41 (4.4)	3.94 ± 2.38	0–12
	≥ 1	895 (95.6)		
Depression	Normal	414 (44.3)	5.89 ± 4.30	0–15
	Depression	522 (55.7)		
Falls (times/year)	None	692 (73.9)	0.52 ± 1.40	0–20
	1	142 (15.2)		
	2	48 (5.2)		
	≥ 3	54 (5.7)		
ED visits (times/year)	None	831 (88.8)	0.16 ± 0.66	0–14
	1	82 (8.8)		
	2	14 (1.5)		
	≥ 3	9 (0.9)		
**Social characteristics**				
Participation in social activity	≥ 3–4 times/week	425 (45.4)		
	≥ 1–2 times/week	218 (23.3)		
	≥ 1–2 times/month	43 (4.6)		
	None	250 (26.7)		
Interactions with family members	≥ 1–2 times/week	438 (46.9)		
	≥ 1–2 times/month	225 (24.0)		
	≥ 1–2 times/quarter	75 (8.0)		
	≥ 1–2 times/year	89 (9.5)		
	None	109 (11.6)		
Interactions with neighbours	≥ 1–2 times/week	698 (74.6)		
	≥ 1–2 times/month	97 (10.4)		
	≥ 1–2 times/quarter	15 (1.6)		
	≥ 1–2 times/year	19 (2.0)		
	None	107 (11.4)		
Public service utilisation	Yes	423 (45.2)		
	No	513 (54.8)		
Social support	Good	202 (21.6)	3.71 ± 1.94	0–6
	Fair	349 (37.3)		
	Poor	385 (41.1)		
Loneliness	High	71 (7.6)	41.89 ± 13.57	20–80
	Moderately high	189 (20.2)		
	Moderate	342 (36.5)		
	Low	334 (35.7)		

*SD* Standard deviation, *ED* Emergency department

Regarding social characteristics, 73.3% participated in social activities, and 45.4% actively participated three to four times a week. Approximately 88% of the participants were intercommunicating with family or neighbours more than once or twice a year. In particular, the rates of active communication with family members and neighbours at least once a week were 46.9 and 74.6%, respectively. Approximately 45.2% received public services, 41.1% lacked social support, and 64.3% felt moderate to high loneliness.

### Factors affecting self-confidence to live alone

Categorical and continuous variables were analysed to identify the relationship between self-confidence to live alone and each of the variables, without controlling for covariates (Tables 3 and 4). As shown in Table 3, in terms of general characteristics, participants with a high-level educational background, economically active participants, and participants who were not exposed to wall cracks or mould at home had higher confidence in living alone than their counterparts. Regarding health-related characteristics, IADL-independent and non-depressed participants showed more self-confidence in living alone. In addition, participants who were involved in social activities, who communicated with their family members or neighbours, who did not receive public services, who had high social support, and who felt less loneliness showed more confidence in living alone. Among the continuous variables, income showed a positive correlation with self-confidence to live alone, but the number of comorbidities, fall history, and ED visits were negatively correlated with self-confidence to live alone (Table 4).

**Table 3:** Self-confidence to live alone by the characteristics of participants (*N* = 936)

Variables	Categories	Self-confidence to live alone	
		Mean ± ***SD***	t (*p*)
**General characteristics**			
Age (years)	65–74	6.78 ± 2.92	1.363 (.173)
	≥ 75	6.49 ± 3.05	
Sex	Male	6.40 ± 2.91	1.098 (.272)
	Female	6.65 ± 3.04	
Educational level	< Middle school	6.44 ± 3.14	2.360 (.019)
	≥ Middle school	6.91 ± 2.69	
Religion	Yes	6.61 ± 2.95	0.271 (.787)
	No	6.55 ± 3.13	
Participation in economic activity	< 3–4 times/week	6.40 ± 3.04	3.346 (.001)
	≥ 3–4 times/week	7.15 ± 2.85	
Wall cracks at home	Yes	6.13 ± 3.14	2.370 (.018)
	No	6.71 ± 2.96	
Mould exposure at home	Yes	5.81 ± 3.12	5.242 (< .001)
	No	6.92 ± 2.90	
**Health-related characteristics**			
Disability	Yes	6.38 ± 3.13	1.068 (.286)
	No	6.64 ± 2.98	
IADL	Independent	6.89 ± 2.87	6.618 (< .001)
	Partially or totally dependent	5.26 ± 3.23	
Depression	No	7.96 ± 2.32	14.051 (< .001)
	Yes	5.50 ± 3.05	
**Social characteristics**			
Participation in social activity	< 3–4 times/week	6.30 ± 3.03	3.256 (.001)
	≥ 3–4 times/week	6.94 ± 2.94	
Interactions with family members	< 1–2 times/month	5.78 ± 3.14	5.168 (< .001)
	≥ 1–2 times/month	6.92 ± 2.89	
Interactions with neighbours	< 1–2 times/month	5.71 ± 3.49	3.333 (.001)
	≥ 1–2 times/month	6.75 ± 2.89	
Public service utilisation	None	6.95 ± 2.82	4.062 (< .001)
	≥ 1	6.15 ± 3.17	
Social support	Good or Fair	7.31 ± 2.58	8.760 (< .001)
	Poor	5.56 ± 3.27	
Loneliness	≤ Moderate	7.14 ± 2.69	8.599 (< .001)
	≥ Moderately high	5.16 ± 3.31	

*SD* Standard deviation, *IADL* Instrumental activities of daily living

**Table 4:** Correlations between self-confidence to live alone and other variables (*N* = 936)

Variables	Self-confidence to live alone	Age	Personal income	Number of comorbidities	Falls
Age (years)	−.061 (.062)				
Personal income (KRW/month)	.118 (< .001)	−.214 (< .001)			
Number of comorbidities	−.172 (< .001)	−.018 (.579)	−.063 (.053)		
Falls (times/year)	−.112 (.001)	.005 (.874)	−.032 (.326)	.106 (.001)	
ED visits (times/year)	−.141 (< .001)	−.026 (.418)	−.044 (.178)	.094 (.004)	.051 (.119)

*ED* Emergency department, *KRW* Korean Won

For the regression model, variables with significance levels < .05 in Tables 3 and 4 were selected. The variance inflation factor (VIF) ranged from 1.032 to 1.636 in all models, and the Durbin-Watson statistic was 1.960 for Model 3. Thus, it is supposed that there were no intercorrelations between variables (i.e. VIF <  10) and no statistical evidence of autocorrelation was found (i.e. 1.8 < d < 2.2) [44]. The results of the hierarchical multiple linear regressions are presented in Table 5. Each stage tested the relationship between self-confidence to live alone and the participants’ characteristics step-by-step. All models were significant (*p* < .05); the adjusted *R*^*2*^ value increased from .046 in Model 1 to .244 in Model 3. The change from Model 1 to Model 2 with health-related characteristics was the largest (*Δ R*^*2*^ = .171). In the final regression model, mould exposure at home, IADL, depression, history of ED visits, interactions with family members, social service utilisation, social support, and loneliness were selected as predictive factors for self-confidence to live alone.

**Table 5:** Factors for self-confidence to live alone (*N* = 936)

Variables	Model 1				Model 2				Model 3			
	***B***	***SE***	***ß***	***p***	***B***	***SE***	***ß***	***p***	***B***	***SE***	***ß***	***p***
Constant	4.097	.495		<.001	6.778	.507		<.001	6.502	.616		<.001
**General characteristics**												
Educational level (ref. < middle school)	0.252	.213	.039	.237	0.075	.197	.012	.380	0.082	.196	.013	.677
Personal income (KRW/month)	6.122E-7	< .001	.081	.016	3.056E-7	< .001	.040	.188	2.398E-7	< .001	.032	.294
Participation in economic activity (ref. < 3–4 times/week)	0.656	.225	.095	.004	0.483	.207	.070	.020	0.379	.204	.055	.064
Wall cracks at home (ref. yes)	0.023	.263	−.003	.929	−0.081	.239	−.011	.736	0.005	.236	.001	.983
Mould exposure at home (ref. yes)	1.074	.231	−.163	< .001	0.746	.212	.113	< .001	0.686	.209	.104	.001
**Health-related characteristics**												
IADL (ref. Independent)					−0.981	.235	−.127	< .001	−0.872	.233	−.113	< .001
Number of comorbidities					−0.048	.039	−.038	.215	−0.043	.038	−.034	.264
Depression (ref. normal)					−2.056	.186	−.340	< .001	−1.579	.199	−.261	< .001
Falls (times/year)					−0.128	.063	−.060	.041	−0.100	.062	−.047	.107
ED visit (times/year)					−0.388	.135	−.085	.004	−0.359	.133	−.079	.007
**Social characteristics**												
Participation in social activity (ref. < 3–4 times/week)									0.192	.181	.032	.290
Interactions with family members (ref. < 1–2 times/month)									0.453	.203	.068	.026
Interactions with neighbours (ref. < 1−2 times/month)									−0.282	.277	−.034	.308
Public service utilisation (ref. ≥ 1)									0.415	.178	.069	.020
Social support (ref. Good or Fair)									−0.659	.206	−.108	.001
Loneliness (ref. ≤ Moderate)									−0.590	.244	−.088	.016
*R*^*2*^ (*Δ R*^*2*^)	.051 (.051)				.222 (.171)				.257 (.035)			
*Adjusted R*^*2*^	.046				.213				.244			
*Δ F* (*p*)	9.937 (< .001)				26.347 (< .001)				19.869 (< .001)			

*SE* Standard error, *IADL* Instrumental activities of daily living, *ED* Emergency department, *KRW* Korean Won

## Discussion

In the present study, mould exposure at home, IADL, depression, ED visits, interactions with family members, public service utilisation, social support, and loneliness were significant predictive factors for self-confidence in living alone.

The concept of self-confidence to live alone was introduced for the first time to promote successful AIP among older adults living alone, and the influencing factors were identified by comprehensively considering the characteristics of ageing. Self-confidence is a familiar and handy indicator that can consider various statuses, coping skills, competence, and knowledge [18]. Therefore, the findings of this study may be evidence that self-confidence to live alone could be a new practical index in the field of health and ageing to screen for successful AIP among older adults living alone.

The average self-confidence to live alone was 6.59 points, slightly higher than the medium score of 5. While refraining from drawing hasty conclusions, it is possible to speculate the reasons for the current findings based on a previous study demonstrating that the proportion of vulnerable groups who responded as ‘very confident to live in place’ was higher than that of the non-vulnerable group [45]. Older adults living alone are a relatively vulnerable residential group among all older adults. According to a prospective study, although baseline states of mental health and physical function were lower in older women living alone than in those who live with a spouse, older women living alone were not at risk for declining in functional status longitudinally [46]. It can be assumed that as older adults living alone try to adapt and balance changes by themselves (e.g. functional, social, and environmental changes), they might be able to perceive their potential power affirming control over their own life, which is more important than actualising it for successful AIP [16].

Interaction with family members, public service utilisation, social support, and loneliness were predictive factors in the aspect of social characteristics. Participants in the current study were more isolated from family members than those in a previous study conducted in the United States (mean age = 67 years). The results originated from the properties of single living. Nevertheless, it has several implications in terms of familial interactions being an important factor to instil confidence to live alone among these older adults. First, consistent with a previous study [21], it was identified that family is still an essential social network for healthy AIP, despite older adults living alone being significantly more isolated from family than those living with others [3]. This finding strengthens the importance of familial networks for older adults living alone.

Similarly, a second implication is the health providers’ and policy makers’ need to pay attention to familial networks for solitary living older adults to promote social networks. Despite the importance of familial connectedness, according to a previous study that reviewed interventions for social connectedness, many studies have focussed on improving contacts with groups or individuals rather than with family members (e.g. peer-group support or community-based programmes) [47]. Research evidence shows that technology-assisted interventions improve social connectedness and belongingness [48], and technological strategies are helpful in promoting familial networks for older adults living alone.

Interestingly, those who use public services showed lower self-confidence to live alone in the current study. Contrary to our results, a generous utilisation of welfare and public services was positively associated with both living alone and actualisation of AIP [6, 25]. This difference could be due to shortcomings in the quantity and quality of services, as inferred from previous studies. Solitary living older adults stated the need for public services to live independently [19] but mentioned limited time [19] and provider-oriented services as disadvantages [49].

In addition, this study shows that social support is an important factor in instilling self-confidence for living alone. This can be linked to the results of existing studies that show social support as essential to maintain independent AIP among older adults living alone. In previous qualitative studies, older adults living alone emphasised that informal social supports were crucial sources of emotional as well as instrumental support such as meal preparation, housework, and hospital visits [17, 19]. In particular, these individuals had significantly lower social support and daily housework support when they were sick than those living with others [50]. Therefore, strengthening a community-based social support system can improve self-confidence to live alone and further enhance independent living for older adults living alone. However, one limitation of this cross-sectional research is that this association must be further explored in prospective studies. The negative relationship between loneliness and self-confidence to live alone found in this study is consistent with a previous study that revealed a negative correlation between loneliness and self-esteem mediated by positive coping strategies [51]. In particular, according to a previous study, older adults living alone experienced more emotional loneliness than those living with others [3]. Therefore, reducing the emotional loneliness of older adults living alone and improving strategies for positive coping skills can increase confidence in living alone and can lead to successful AIP.

With respect to health-related factors, IADL and depression were important for self-confidence to live alone as demonstrated in many studies. IADL and depression are factors that share several important aspects of independent living for older adults. According to a previous study on these individuals, the declining capability of IADL increases their difficulties in daily living, such as household affairs, shopping, gardening, and household maintenance, which makes it harder for them to live independently and increases feelings of depression [52, 53]. In other aspects, difficulties in IADL hinder social activities, making older adults depressed, and eventually impairing their sense of mastery over their own life [54]. Therefore, for older adults to live independently, it is necessary to examine those who have difficulties with IADL, to identify domains requiring support in the IADL, and to establish an easily accessible social daily living assistance system. In addition, in-depth evaluation of the causes of depression is required for the elderly living alone with low confidence in AIP, and tailored interventions based on that can lead to successful AIP.

History of ED visits was a significant factor in lowering self-confidence to live alone. Most older adults living alone require emergency support services, especially for safety [55]. This means that people living alone must confront and handle any life-threatening situation without immediate help, which may make them anxious. This experience of helplessness in a life crisis situation can negatively affect self-confidence in living alone and can even lead to dependency upon others. Therefore, reducing the incidence of emergency situations among older adults living alone could increase their self-confidence to live alone.

Finally, exposure to indoor microbial pollutants is a well-known health hazard that causes many symptoms and diseases [23]. It can be assumed that long-term exposure to such an adverse environment leads to chronic pain and decline in health, resulting in poor quality of life and, consequently, reducing the confidence to live alone. Therefore, periodic inspection of the residential conditions of older adults living alone is necessary, alongside health education on the importance of home management methods.

Despite these findings, the current study has some limitations. First, since this study was the first to measure the confidence to live alone, the concept was defined as evidenced in previous studies related to AIP, independent living, and healthy and successful ageing. For a more consistent implementation of the concept in the field, further conceptual analysis is necessary. Second, self-confidence to live alone was measured using a simple and convenient NRS scale considering the characteristics of older adults, however, it is not a structured tool. Thus, future studies need to develop a standardised measurement and present clinimetric properties. Third, this study comprehensively encompasses many variables based on previous studies, but there were limits to exploring related factors in-depth due to secondary data analysis. Further research needs to explore factors with their constituent domains and their pathways. Fourth, though the original data were collected by trained assistants, interpretation of the results should be done with caution as this study could potentially be impacted by self-report bias.

## Conclusions

Self-confidence to live alone—a new and practical indicator for screening successful AIP among older adults living alone—was slightly higher than the mean value of the scale. To increase self-confidence to live alone, intervention measures must focus on improving interactions with family members, public service utilisation, social support, loneliness, IADL, depression, ED visits, and mould exposure at home. The findings have scope for fostering independent living and successful AIP among older adults living alone.

## Data Availability

The data used and/or analysed during the current study are available from the corresponding author on reasonable request.

## Abbreviations

AIP: Ageing in place
ED: Emergency Department
ENRICHD: Enhancing recovery in coronary health disease
ESSI: ENRICHD social support instrument
K-IADL: Korean instrumental activities of daily living
KRW: Korean Won
MMSE: Mini-mental status examination
NRS: Numeric rating scale
SD: Standard deviation
SE: Standard error
VIF: Variance inflation factor

## References

[1] Reher D, Requena M. Living alone in later life: a global perspective. Popul Dev Rev. 2018;44(3):427–54. 10.1111/padr.12149.

[2] Statistics Korea. Population projection for Korea. 2019. https://kosis.kr/statHtml/statHtml.do?orgId=101&tblId=DT_1BZ0503&vw_cd=MT_ZTITLE&list_id=A42_10&seqNo=&lang_mode=ko&language=kor&obj_var_id=&itm_id=&conn_path=MT_ZTITLE. Accessed 10 October 2020.

[3] Evans IEM, Llewellyn DJ, Matthews FE, Woods RT, Brayne C, Clare L, et al. Living alone and cognitive function in later life. Arch Gerontol Geriatr. 2019;81:222–33. 10.1016/j.archger.2018.12.014. 10.1016/j.archger.2018.12.014 30654180

[4] Roh M, Weon S. Living arrangement and life satisfaction of the elderly in South Korea. Soc Indic Res [Internet]. 2020. 10.1007/s11205-020-02443-3. Accessed 10 October 2020.

[5] Srugo SA, Jiang Y, de Groh M. At-a-glance - Living arrangements and health status of seniors in the 2018 Canadian Community Health Survey. Health Promot Chronic Dis Prev Can. 2020;40(1):18–22. 10.24095/hpcdp.40.1.03. 10.24095/hpcdp.40.1.03 PMC7051173 31939634

[6] Kendig H, Gong CH, Cannon L, Browning C. Preferences and predictors of aging in place: longitudinal evidence from Melbourne. Australia. J Hous Elderly. 2017;31(3):259–71. 10.1080/02763893.2017.1280582.

[7] Korea Institute for Health and Social Affairs. 2017 survey of living conditions and welfare needs of Korean older persons. 2018. https://www.kihasa.re.kr/web/publication/research/view.do?menuId=45&tid=71&bid=12&division=002&ano=2298. Accessed 10 October 2020.

[8] Bigonnesse C, Chaudhury H. The landscape of “aging in place” in gerontology literature: emergence, theoretical perspectives, and influencing factors. J Aging Environ. 2020;34(3):233–51. 10.1080/02763893.2019.1638875.

[9] WHO Centre for Health Development (Kobe, Japan). A glossary of terms for community health care and services for older persons. Kobe: WHO Centre for Health Development; 2004. https://apps.who.int/iris/handle/10665/68896. Accessed 10 October 2020.

[10] Ahn M. Introduction to special issue: aging in place. Hous Soc. 2017;44(1–2):1–3. 10.1080/08882746.2017.1398450.

[11] Stones D, Gullifer J. ‘At home it’s just so much easier to be yourself’: older adults’ perceptions of ageing in place. Ageing Soc. 2016;36(3):449–81. 10.1017/s0144686x14001214.

[12] Torres-Gil F, Hofland B. Vulnerable populations. In H. Cisneros, M. Dyer-Chamberlain, & J. Hickies (Eds.), Independent for life: Homes and neighborhoods for an aging America. Austin: University of Texas Press; 2012. p. 221–32.

[13] Hillcoat-Nallétamby S. The meaning of “independence” for older people in different residential settings. J Gerontol B Psychol Sci Soc Sci. 2014;69(3):419–430. 10.1093/geronb/gbu008 24578371

[14] Mirowsky J. Age and the sense of control. Soc Psychol Q. 1995;58(1):31. 10.2307/2787141.

[15] Lawton MP. The elderly in context: perspectives from environmental psychology and gerontology. Environ Behav. 1985;17(4):501–19. 10.1177/0013916585174005.

[16] Pirhonen J, Pietilä I. Perceived resident-facility fit and sense of control in assisted living. J Aging Stud. 2016;38:47–56. 10.1016/j.jaging.2016.04.006. 10.1016/j.jaging.2016.04.006 27531452

[17] Dupuis-Blanchard S, Gould ON, Gibbons C, Simard M, Éthier S, Villalon L. Strategies for aging in place: the experience of language-minority seniors with loss of independence. Glob Qual Nurs Res. 2015;(2):1–9. 10.1177/2333393614565187. 10.1177/2333393614565187 PMC5342846 28462299

[18] Moore BM (1952). Self-confidence for competence. Edu Leader.

[19] Narushima M, Kawabata M. “Fiercely independent”: experiences of aging in the right place of older women living alone with physical limitations. J Aging Stud. 2020;54:100875. 10.1016/j.jaging.2020.100875. 10.1016/j.jaging.2020.100875 PMC7480772 32972619

[20] Bacsu J, Jeffery B, Abonyi S, Johnson S, Novik N, Martz D, et al. Healthy aging in place: perceptions of rural older adults. Educ Gerontol. 2014;40(5):327–37. 10.1080/03601277.2013.802191.

[21] Li B, Chen S. A study of residential condition and satisfaction of the elderly in China. J Hous Elderly. 2011;25(1):72–88. 10.1080/02763893.2011.545746.

[22] World Health Organization Regional Office for Europe. WHO guidelines for indoor air quality: dampness and mould [Internet]. 2009. https://www.euro.who.int/__data/assets/pdf_file/0017/43325/E92645.pdf. Accessed 10 October 2020. 23785740

[23] Fernández-Carro C, Módenes JA, Spijker J. Living conditions as predictor of elderly residential satisfaction. A cross-European view by poverty status. Eur J Ageing. 2015;12(3):187–202. 10.1007/s10433-015-0338-z. 10.1007/s10433-015-0338-z PMC5549235 28804354

[24] Lee YS, Kim YS, Sung CH, Shin YJ, Cho WS. A descriptive research on field situation of customized modification for vulnerable single elderly home. J Korean Hous Assoc. 2017;28(3):55–64. 10.6107/jkha.2017.28.3.055.

[25] Mudrazija S, Angel J, Cipin I, Smolic S. Living alone in the United States and Europe: the impact of public support on the independence of older adults. Innov Aging. 2018;2(suppl_1):769–70. 10.1093/geroni/igy023.2849. 10.1177/0164027520907332 PMC10782838 32116111

[26] Park S, Han Y, Kim B, Dunkle RE. Aging in place of vulnerable older adults: person-environment fit perspective. J Appl Gerontol. 2017;36(11):1327–50. 10.1177/0733464815617286. 10.1177/0733464815617286 26610906

[27] Falls. World Health Organization. 2018. https://www.who.int/news-room/fact-sheets/detail/falls. Accessed 10 October 2020.

[28] Choi NG, Choi BY, DiNitto DM, Marti CN, Kunik ME. Fall-related emergency department visits and hospitalizations among community-dwelling older adults: examination of health problems and injury characteristics. BMC Geriatr. 2019;19(1):303. 10.1186/s12877-019-1329-2. 10.1186/s12877-019-1329-2 PMC6849272 31711437

[29] Dingová M, Králová E. Fear of falling among community dwelling older adults. Cent Eur J Nurs Midwifery. 2017;8(1):580–7. 10.15452/cejnm.2017.08.0005.

[30] Lau DT, Scandrett KG, Jarzebowski M, Holman K, Emanuel L. Health-related safety: a framework to address barriers to aging in place. Gerontologist. 2007;47(6):830–7. 10.1093/geront/47.6.830. 10.1093/geront/47.6.830 18192636

[31] Care service for older adults. Ministry of Health Welfare. 2017. https://www.mohw.go.kr/react/jb/sjb030301vw.jsp.PAR_MENU_ID=03&MENU_ID=032901&CONT_SEQ=338646. Accessed 10 Oct 2020.

[32] Won CW, Yang KY, Rho YG, Kim SY, Lee EJ, Yoon JL, et al. The development of Korean activities of daily living (K-ADL) and Korean instrumental activities of daily living (K-IADL) scale. J Korean Geriatr Soc. 2002; 6(2): 107–120. https://www.e-agmr.org/journal/view.php?number=280. Accessed 10 October 2020.

[33] Lee HS, Kim HS, Jung YM. Depression and quality of life in Korean elders. J Korean Acad Commun Health Nurs. 2009;20(1):12–22. https://www.koreascience.or.kr/article/JAKO200916263468652.pdf. Accessed 7 Mar 2021.

[34] Won CW, Rho YG, Sunwoo D, Lee YS. The validity and reliability of Korean Instrumental Activities of Daily Living (K-IADL) scale. J Korean Geriatr Soc. 2002; 6(4): 273–280. https://www.e-agmr.org/journal/view.php?vol=6&issue=4&spage=273. Accessed 7 March 2021.

[35] Kee BS. A preliminary study for the standardization of geriatric depression scale short form-Korea version. J Korean Neuropsychiatr Assoc*.* 1996; 35(2): 298–307. https://kmbase.medric.or.kr/Main.aspx?menu=01&d=KMBASE&m=VIEW&i=0368819960350020298. Accessed 10 Oct 2020.

[36] Sheikh JI, Yesavage JA. Geriatric depression scale (GDS): recent evidence and development of a shorter version. Clin Gerontol. 1986;5(1–2):165–73. 10.1300/J018v05n01_09.

[37] Mortimer JA, Ding D, Borenstein AR, DeCarli C, Guo Q, Wu Y, et al. Changes in brain volume and cognition in a randomized trial of exercise and social interaction in a community-based sample of non-demented Chinese elders. J Alzheimers Dis. 2012;30(4):757–66. 10.3233/JAD-2012-120079. 10.3233/JAD-2012-120079 PMC3788823 22451320

[38] Shankar A, McMunn A, Banks J, Steptoe A. Loneliness, social isolation, and behavioral and biological health indicators in older adults. Health Psychol. 2011;30(4):377–85. 10.1037/a0022826. 10.1037/a0022826 21534675

[39] Mitchell PH, Powell L, Blumenthal J, Norten J, Ironson G, Pitula CR, et al. A short social support measure for patients recovering from myocardial infarction: the enriched social support inventory. J Cardiopulm Rehabil. 2003;23(6):398–403. 10.1097/00008483-200311000-00001. 10.1097/00008483-200311000-00001 14646785

[40] Jeon GS, Jang SN, Park S. Social support, social network, and frailty in Korean elderly. J Korean Geriatr Soc. 2012;16(2):84–94. 10.4235/jkgs.2012.16.2.84.

[41] Kim OS. Korean version of the revised UCLA loneliness scale: reliability and validity test. Kanho Hakhoe Chi. 1997;27(4):871–9. 10.4040/jnas.1997.27.4.871.

[42] Russell D, Peplau LA, Cutrona CE. The revised UCLA Loneliness Scale: concurrent and discriminant validity evidence. J Pers Soc Psychol. 1980;39(3):472–80. 10.1037/0022-3514.39.3.472. 10.1037//0022-3514.39.3.472 7431205

[43] Perry GR (1990). Loneliness and coping among tertiary-level adult cancer patients in the home. Cancer Nurs..

[44] Lee IH. Easyflow regression analysis. Seoul: Hannarae; 2016. p. 313–6.

[45] Tang F, Lee Y. Home- and community-based services utilization and aging in place. Home Health Care Serv Q. 2010;29(3):138–54. 10.1080/01621424.2010.511518. 10.1080/01621424.2010.511518 20845175

[46] Michael YL, Berkman LF, Colditz GA, Kawachi I (2001). Living arrangements, social integration, and change in functional health status. Am J Epidemiol..

[47] O’Rourke HM, Collins L, Sidani S. Interventions to address social connectedness and loneliness for older adults: a scoping review. BMC Geriatr. 2018;18(1):214. 10.1186/s12877-018-0897-x. 10.1186/s12877-018-0897-x PMC6139173 30219034

[48] Stojanovic J, Collamati A, Duplaga M, Onder G, La Milia DI, Ricciardi W, et al. Decreasing loneliness and social isolation among the older people: systematic search and narrative review. Epidemiol Biostat Public Health. 2017;14(suppl 1):2. 10.2427/12408.

[49] Park YH, Lim KC, Cho BL, Ko H, Yi YM, Noh EY, et al. Experiences of healthcare and daily life support services in community-dwelling elders living alone: a thematic analysis using focus group interviewing. J Korean Gerontol Nurs. 2019;21(3):200–10. 10.17079/jkgn.2019.21.3.200.

[50] Chan E, Procter-Gray E, Churchill L, Cheng J, Siden R, et al. Associations among living alone, social support and social activity in older adults. AIMS Public Health. 2020;7(3):521–34. 10.3934/publichealth.2020042. 10.3934/publichealth.2020042 PMC7505797 32968675

[51] Zhao L, Zhang X, Ran G. Positive coping style as a mediator between older adults’ self-esteem and loneliness. Soc Behav Pers. 2017;45(10):1619–28. 10.2224/sbp.6486.

[52] Ahlqvist A, Nyfors H, Suhonen R. Factors associated with older people’s independent living from the viewpoint of health and functional capacity: a register-based study. Nurs Open. 2016;3(2):79–89. 10.1002/nop2.39. 10.1002/nop2.39 PMC5047332 27708818

[53] Stahl ST, Beach SR, Musa D, Schulz R. Living alone and depression: the modifying role of the perceived neighborhood environment. Aging Ment Health. 2017;21(10):1065–71. 10.1080/13607863.2016.1191060. 10.1080/13607863.2016.1191060 PMC5161727 27267633

[54] Lin IF, Wu HS. Does informal care attenuate the cycle of ADL/IADL disability and depressive symptoms in late life? J Gerontol B Psychol Sci Soc Sci. 2011;66(5):585–94. 10.1093/geronb/gbr060. 10.1093/geronb/gbr060 PMC3155031 21746870

[55] Ko H, Park YH, Cho B, Lim KC, Chang SJ, Yi YM, et al. Gender differences in health status, quality of life, and community service needs of older adults living alone. Arch Gerontol Geriatr. 2019;83:239–45. 10.1016/j.archger.2019.05.009. 10.1016/j.archger.2019.05.009 31102926

